# Chinese Medicine Injection Qingkailing for Treatment of Acute Ischemia Stroke: A Systematic Review of Randomized Controlled Trials

**DOI:** 10.1155/2012/213172

**Published:** 2012-05-22

**Authors:** Fafeng Cheng, Xueqian Wang, Yi Lu, Xianggen Zhong, Yan Zhao, Qingguo Wang

**Affiliations:** College of Basic Medicine, Beijing University of Chinese Medicine, Beijing 100029, China

## Abstract

Qingkailing (QKL) injection was a famous traditional Chinese patent medicine, which was extensively used to treat the acute stages of cerebrovascular disease. The aim of this study was to assess the quantity, quality and overall strength of the evidence on QKL in the treatment of acute ischemic stroke. *Methods*. An extensive search was performed within MEDLINE, Cochrane, CNKI, Vip and Wan-Fang up to November 2011. Randomized controlled trails (RCTs) on QKL for treatment of acute stroke were collected, irrespective of languages. Study selection, data extraction, quality assessment, and data analyses were conducted according to the Cochrane standards, and RevMan5 was used for data analysis. *Results*. 7 RCTs (545 patients) were included and the methodological quality was evaluated as generally low. The pooled results showed that QKL combined with conventional treatment was more effective in effect rate, and the score of MESSS and TNF-**α** level compared with conventional treatment alone, but there was no significant difference in mortality of two groups. Only one trial reported routine life status. There were four trials reported adverse events, and no obvious adverse event occurred in three trials while one reported adverse events described as eruption and dizziness.

## 1. Introduction

Stroke is a major cause of death and disability in the world and the most important strategy for treatment of ischemic stroke is prevention and effective therapy. In China, acupuncture and traditional Chinese medicine (TCM) have been used to treat stroke patent medicine or a history over 2000 years. In recent decades, patent medicines of TCM were widely and regularly used in stroke patients in either Western medicine hospitals or traditional Chinese medicine hospitals. However, few studies have been published in English reporting the effectiveness and safety of many commonly used TCM [1, 2]. Currently, there is yet no routine effective, generally accepted, specific treatment for ischemic stroke, except for thrombolytic treatment for highly selected patients. Therefore, confirmation of the effectiveness of TCM could have a great impact on stroke management in the world.

Qingkailing (QKL) injection was originally prepared by the Beijing University of Chinese Medicine in the 1970s, by modifying a traditional Chinese medicine, Angongniuhuang pills, composed of *Radix Isatidis*, *Flos Lonicerae*, *Concha Margaritifera Usta*, baicalin, *Fructus gardeniae*, cholic acid, hyodeoxycholic acid, and *Cornu Bubali* [3]. It has been extensively used to treat the acute stages of cerebrovascular disease and has performed excellently in improving neurological function [4]. Animal experiments have shown that QKL injection can promote endothelial nitric oxide synthase expression, reduce calcium overload, regulate matrix metalloproteinase-9 expression, and inhibit inflammation in a murine model of cerebral ischemia/reperfusion [5–8]. Several clinical studies reported the effectiveness ranging from case reports and randomized clinical trials, but the evidence for its effect was not clear.

The aim of this study was to assess the quantity, quality and overall strength of the evidence on QKL in the treatment of acute ischemic stroke.

## 2. Methods

### 2.1. Database and Search Strategies

The electronic databases of MEDLINE (1982–2011), Cochrane Controlled Trials Register ((Issue 10, 2011)), CNKI database (1974–2011), Vip database (1989–2011), and Wan-Fang database (1998–2011) were searched by using a combination of MESH subject headings of “Qingkailing” and “Stroke” or “cerebral infarction” without language limitation. Reference lists from trials selected by electronic searching and conference compilations were hand searched. All of those searches ended before November 2011.

### 2.2. Inclusion Criteria

Definite or possible randomized controlled trials (RCTs) were included. RCTs combined QKL injection with conventional treatment with the conventional treatment alone, and QKL injection plus conventional treatment compared with a western medicine plus conventional treatment were included. While the trials with other TCM used either in QKL or control groups were excluded. There were no restrictions on population characteristics, language, and publication type.

 Trials that included patients of any age or sex with acute ischemic stroke were eligible. Ischemic stroke was defined if the patients met the World Health Organization or the similar Chinese National criteria (demanding a CT/MRI scan as confirmation) of stroke, and hemorrhagic stroke was excluded. Possible ischemic stroke in which CT/MRI were not performed was also to be included.

The primary outcome measures were death and effect rate (which was based on the neurological deficit improvement), and adverse events. The secondary outcome measures were neurological deficit scoring, infarction volume of CT, inflammation factors, and quality of life.

### 2.3. Data Extraction and Quality Assessment

The data was entered into an electronic database by the two reviewers (F. F. Cheng and X. Q. Wang) independently, in the case where the two entries did not match, and a third person (Y. Lu) may be involved for verification. Quality assessment of included randomized controlled trials: sequence generation, allocation concealment, blinding of participants personnel and outcome assessors, incomplete outcome data, selective outcome reporting, and other sources of bias [9].

### 2.4. Data Synthesis

The statistical package (RevMan 5) was used for data analyses, which was provided by the Cochrane Collaboration. Dichotomous data were presented as risk ratio (RR) and continuous outcomes as mean difference (MD), both with 95% confidence interval (CI). Heterogeneity between trials results was tested, and heterogeneity was presented as significant when *I* ^2^ is over 50% or *P* < 0.1. Random effect model was used for the meta-analysis if there was significant heterogeneity and fixed effect model was used when the heterogeneity was not significant [9]. Publication bias was explored via a funnel-plot analysis.

## 3. Result

### 3.1. Description of Included Trials

**Figure 1:**
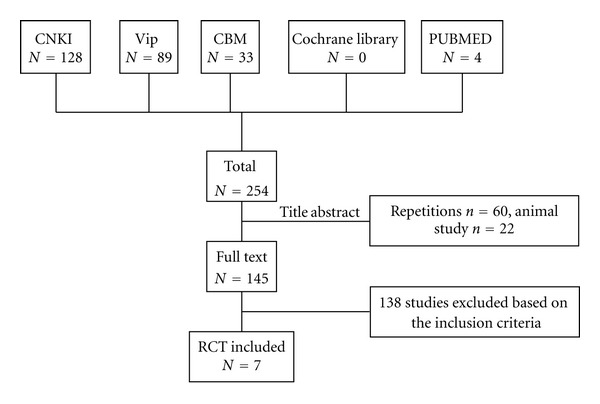
Study selection process.

After primary search of 5 databases, 254 trials were screened out from electronic and manual searches (Figure 1), and the majority were excluded due to being found from more than one database or obvious ineligibility which including irrelevant titles and abstract. 6 trials were excluded because of duplicated publication, 62 trials were excluded due to the animal studies, the rest 41 trials were noncontrolled clinical trials including case report, case series, or review. 145 full text papers were retrieved and 138 trials were excluded based on the inclusion criteria. The most frequent reasons for exclusion were other types of intervention examined (78), either no proper outcomes reported (29), or not a randomized trial (24), while 7 RCTs including cerebral hemorrhage were excluded. In the end, 7 RCTs (545 participants) [10–16] were reviewed. All studies were published in Chinese. The bibliographic details of these trials are given in Table 1.

The age of patients in the included studies ranged from 33 to 78 years old. All trials included more males (60% to 70%) than females. All included trials applied standard medicine diagnostic criteria for ischemia stroke, and all of them reported the need for all patients to have had CT/MRI scanning to confirm the diagnosis. Only QKL injection was studied in these included trials. The dose range of QKL injection was from 30 mL to 80 mL and dose route was intravenous injection in all included studies. Most of included trials were designed to compared QKL injection plus conventional treatment with the conventional treatment alone, the later including basic therapy, and urokinase, and only one trail [10] compared QKL injection plus conventional treatment with nimodipine plus conventional treatment (Table 1). None of the trials was randomized, double-blind, placebo controlled. The timing of the start of treatment after stroke onset was within 72 hours, and the total duration of treatment varied from 7 to 20 days.

All the including trials reported the effect rate, but only two mentioned the death rate. Only one trial [11] had undergone assessment of patients' routine living ability after treatment. Three trials [11–13] reported neurological deficit, using the Modified Edinburgh-Scandinavian Stroke Scale (MESSS), which was recommended at the Second and revised at the Fourth National Cerebrovascular Diseases Conference in China. And one trial [12] measured infarction volume of CT. Tan et al. [10] and Wu et al. [14] reported inflammation factor in blood serum, both including TNF-*α* and IL-6. Yang et al. [12] reported the effect of QKL injection in treating coma induced by stroke.

### 3.2. Methodological Quality of Included Trials

The methodological quality of most included trials was generally “poor.” The sample size of including trials varied from 40 to 150 patients. None of the 26 trials reported sample size calculation. None of the trials was properly randomized, double blinded, placebo controlled, and no trials employed a blinding procedure, yet only one trial reported the method of randomization (random number table) [10]. None of the trials had grade A level of adequate concealment of randomization. None of the trials reported the number of patients that were lost to followup and whether they had used intention-to-treat analysis (Table 2).

### 3.3. Effect of the Interventions

#### 3.3.1. Primary Outcomes

**Effect Rate**

**Figure 2:**
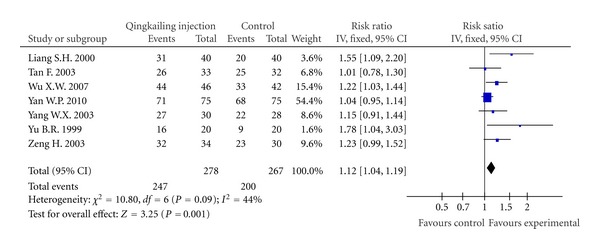
Forest plot of comparison: effect rate.

All the seven trials reported clinical effect rate to evaluate the outcome, which was based on the reduction of neurological deficit scoring [18]. Four trails [11–14] used the percentage of MESSS scores reduced rate to measure the outcome: cure (MESSS scores reduced rate from 91 to 100%), significantly effective (MESSS scores reduced rate more from 46 to 90%), effective (MESSS scores reduced rate from 18 to 46%), and ineffective (MESSS scores reduced rate less than 18%). Other three studies [10, 15, 16] used similar evaluation standards at the study time. We put these two different kinds of measurements together to evaluate the general effectiveness. Total effective rate is the combination of cure, significant effective and effective rate. The meta-analysis showed significant difference between groups of QKL injection plus conventional treatment and conventional treatment alone on total effective rate (RR: 1.12 [1.04, 1.19]; *P* < 0.01) (Figure 2).

**Death**

**Figure 3:**
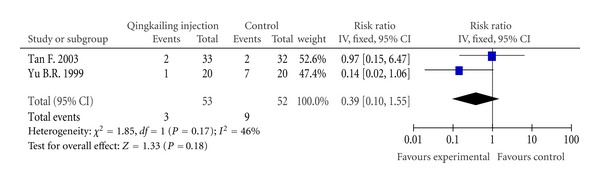
Forest plot of comparison: death.

Two trials [10, 16] out of seven reported death. Tan et al. [10] assessed death at the end of 2-week therapeutic course, and Yu and Liao [16] reported it 1 month after stroke onset. Total number of death was 12 out of 105 patients. There was no statistically significant difference between two groups (RR, 0.39; 95% CI, 0.10 to 1.55; *P* = 0.18) (Figure 3).

**Adverse Effect**

Four trials [10, 12, 13, 16] reported adverse events, while the other three did not mention it. No obvious adverse events occurred in three trials, and one trial [10] reported adverse events described as eruption and dizziness; however, eruption also appeared in control group. The duration of treatment in all included studies was short (7 to 20 days). Outcomes were measured within 1 month after stroke onset, without longer followup. Although few related information about adverse effects was recorded, potential or long-time adverse effects in the included trials could not be excluded (Table 1).

#### 3.3.2. Secondary Outcomes

**MESSS Scoring**

**Figure 4:**
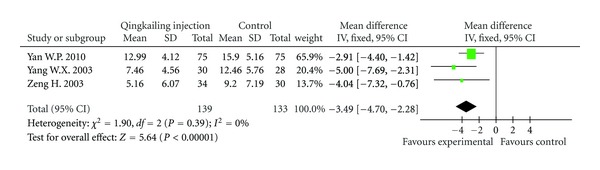
Forest plot of comparison: MESSS scoring.

Three trials (272 patients) [11–13] reported detailed neurological deficit scoring (MESSS). The scoring performed no significant difference between two groups before treatment, and meta-analysis of data on MESSS scoring at the end of duration of treatment showed that QKL injection plus conventional treatment had more benefit compared with conventional treatment alone (WMD: −3.49; 95% CI, −4.70 to −2.28; *P* < 0.01) (Figure 4).

**Infarction Volume**

One trial [12] assessed infarction volume of CT, before treatment and at the end of 21 days of treatment, respectively, using the formula of quantitative estimation on hematoma. The baseline of infarction volume had no significant difference between two groups and the infarction volume of QKL group at endpoint reduced significantly more than control (WMD, −1.85; 95% CI, −3.40 to −0.30; *P* = 0.02).

**Inflammation Factor**

**Figure 5:**
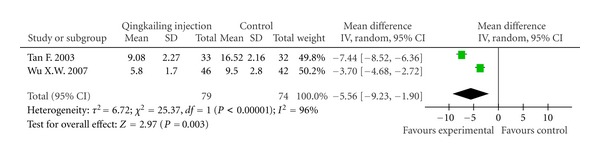
Forest plot of comparison: TNF-*α*.

Two trials [10, 14] provided data of inflammation factors in blood serum. They both reported TNF-*α* and IL-6, so we make meta-analysis of them. At the end of treatment, results showed there was significant difference between QKL treatment and routine treatment groups for TNF-*α* level (WMD −5.56; 95% CI, −9.23 to −1.90; *P* < 0.01), but no statistic significance for IL-6 (WMD −22.61; 95% CI, −53.91 to 8.68; *P* = 0.16) (Figure 5).

**Revival of Coma**

Yang et al. [12] reported the effect of QKL injection in reviving coma induced by stroke. In QKL injection treatment group, 11 coma cases out of 13 revived within 7 days, while in control group that was only 4 out of 10 (RR, 2.12; 95% CI, 0.96 to 4.68; *P* = 0.06).

#### 3.3.3. Quality of Life

Only one trial [11] reported routine life status using scoring recommended at the Second and revised at the Fourth National Cerebrovascular Diseases Conference in China [18]. At the end of treatment, QKL treatment significantly improved routine living ability than control group (WMD, −0.96; 95% CI, −1.84 to 0.08; **P** = 0.03).

### 3.4. Publication Bias

Although we conducted comprehensive searches and tried to avoid bias, since all trials were published in Chinese, we could not exclude potential publication bias.

## 4. Discussion

This review of randomized trials showed the current evidence in QKL injection for acute ischemia stroke. In many years, Western medicine has made tremendous progress and had become the dominating medical treatment worldwide. However, it has been increasingly recognized that Western medicine may sometimes fail to treat an illness, whereas such illness is reportedly improved by the so-called complementary medicine based on a different theory [19, 20]. Traditional Chinese patent medicine (TCPM) for stroke is very popular in China [1, 2, 17]. Currently, there are more than 100 TCPM used for stroke and approved by the Chinese State Food and Drug Administration [17]. QKL injection has been applied for more than 30 years. However, few relevant articles on QKL injection for stroke have been published in the English medical journals. One systemic review about TCPM for ischemic stroke published in 2007 included only 2 trials of QKL injection [17]. One of them was included in this study, while the other excluded for not meeting inclusion criteria.

The data from the 7 RCTs that were analyzed demonstrated that, QKL injection plus conventional medication maybe more effective than conventional medication alone for acute ischemia stroke. With evaluating the improvement of classification of neurological function using MESSS scoring (RR, 1.12; 95% CI, 1.04 to 1.19), the effect rate of QKL plus conventional medicine treatment group was, on average, 14 percent more than control group using conventional medicine alone. In consistency with it, meta-analysis of detailed neurological deficit scoring (MESSS) in three trails also showed QKL group had more benefit in improving neurological function. These results were positively encouraging and promising of combining QKL injection with conventional treatment, which might be beneficial to acute ischemic stroke.

However, there were several limitations in this paper. No multicenter, large sample and cooperative studies were found and most of the existing trials were of small size, yet no trials estimated the sample size. Although all trials claimed randomization, most of them failed to provide enough information to judge whether the randomization procedures had been carried out properly. And inadequate reporting of allocation concealment, blinding, intention to treat analysis, and dropouts account in all the trials may have created potential performance biases and detection biases, as patients and researchers might have been aware of the therapeutic interventions. So every trial had an unclear risk of bias or a high risk of bias. Although we conducted comprehensive searches and tried to avoid bias, since all trials were published in Chinese, there remained the possible existence of publication bias.

This paper showed a very low case fatality rate (12 deaths out of total 547 patients). There were several possible explanations for the striking finding: truly low case fatality rate for stroke in China; severe stroke patients were not sent to hospitals; severe stroke cases were excluded by Chinese stroke physicians in research studies; failure to report major outcome events; or did not assess long-term outcome after stroke onset. Those all resulted in bias. However, in the two trials reporting death with limited sample size, there was a tendency of reducing fatality rate in QKL injection group without significant difference.

For secondary outcomes, infarction volumes of CT and inflammation factors in blood serum were reported in a few trials. These results supported QKL injection's efficiency, and the later outcome was consistent with animal results about the mechanism of QKL in ischemic model [6, 7]. Except for one trial, most of trials did not mention quality of life, which possibly was special advantage of TCM. We suggest future trials for QKL to pay more attention to outcome of life quality and comply with international standards in the evaluation of life status. One trial reported effect in reviving stroke-induced coma and result showed more coma cases in QKL group regained consciousness within one week; however, with no statistic significance because of limited sample. It indicated that QKL may have excellent effect in restoring consciousness, which was called consciousness restoring and obstruction clearing (Xingnaokaiqiao) in TCM theory. Angongniuhuang pill was well known about its excellent consciousness restoring and obstruction clearing effect, and QKL also was widely used to treat coma. So we advise future RCTs to assess QKL effect for subgroup of severe stroke patients combined with coma.

Adverse effects were reported by Tan et al. [10] as eruption and dizziness. No obvious adverse events occurred in three trials, while other three trials did not report adverse effects. However, the concrete conclusion regarding safety cannot be determined from this paper due to the limited evidence provided by the eligible trials. Another paper about the safety of QKL concluded that, although some cases of adverse effects for QKL were reported, QKL carries a low risk of adverse drug reactions and adverse events, and some adverse events that do occur may be ascribed to improper use of the drug [21]. In order to proper assess the safety of QKL injection, large-scale clinical trials with long-term followup are required.

In conclusion, a definite conclusion on efficacy and adverse events associated with QKL injection cannot be drawn from this paper because of the unclear methodological quality of these included trials. The general lack of reporting of methodology in these trials publication was not consistent with the CONSORT statement on the reporting of the results of randomized trials (http://www.consort-statement.org/), which is being highlighted in many journals around the world. Future trials should overcome the limitations of the trials presented in this paper; particularly, they should assure adequate concealment of allocation and blinding of outcome assessors and report both of fatality rate and effect rate as the primary outcomes at long-term followup, and evaluate routine living ability using international standards. If the positive effects of QKL injection were confirmed by comprehensive clinical trials, it would lead to many promising treatments for acute ischemic stroke and could benefit patients all over the world.

## Figures and Tables

**Table 1:** Characteristics and methodological quality of included studies.

Study ID	Sample	Time of onset	CT/MRI	Intervention in control group	QKL injection dose/day	Course	Followup (month)	Death	Adverse effect
Liang 2000 [15]	80	<72 h	Yes	Conventional treatment	30 mL	14 d	1	No	Unclear
Tan et al. 2003 [10]	65	<72 h	Yes	Conventional treatment	40 mL	14 d	No	2/2	2 eruption and 1 dizziness
Wu et al. 2007 [14]	88	<72 h	Yes	Conventional treatment	1200 mg (freeze-drying agent, roughly equivalent to 60 mL)	14 d	No	No	Unclear
Yan and Li 2010 [13]	150	<6 h	Yes	Urokinase + conventional treatment	40 mL	7 d	No	No	No
Yang et al. 2003 [12]	58	<72 h	Yes	Conventional treatment	80 mL	20 d	No	No	No
Zeng and Feng 2003 [11]	64	<24 h	Yes	Conventional treatment	60 mL	20 d	No	No	Unclear
Yu and Liao 1999 [16]	40	<72 h	Yes	Conventional treatment	50 mL	14 d	1	1/7	No

Conventional medicine treatment includes mannital, dextran, nimodipine, aspirin, and so on.

**Table 2:** Quality assessment of included randomized controlled trials.

Study ID	Sequence generation	Allocation concealment	Incomplete outcome data	Blinding	Other source of bias	Selective outcome reporting	Risk of bias
Liang 2000 [15]	Unclear	Unclear	No	Unclear	Unclear	No	Unclear
Tan et al. 2003 [10]	Table of random number	Unclear	Yes	Unclear	Unclear	No	Unclear
Wu et al. 2007 [17]	Unclear	Unclear	No	Unclear	Unclear	No	Unclear
Yan and Li 2010 [13]	Unclear	Unclear	Yes	Unclear	Unclear	Yes	Unclear
Yang et al. 2003 [12]	Unclear	Unclear	No	Unclear	Unclear	No	Unclear
Zeng and Feng 2003 [11]	Unclear	Unclear	No	Unclear	Unclear	No	Unclear
Yu and Liao 1999 [16]	Unclear	Unclear	Yes	Unclear	Unclear	No	Unclear

## References

[1] Wu B, Liu M, Zhang S (2004). Dan Shen agents for acute ischaemic stroke. *Cochrane Database of Systematic Reviews*.

[2] Zeng X, Liu M, Yang Y, Li Y, Asplund K (2006). Ginkgo biloba for acute ischemic stroke. *Stroke*.

[3] Beijing University of Chinese Medicine (1975). The study of novel dosage form of An Gong Niu Huang Wan. *Journal of New Medicine*.

[4] Cheng FF, Song WT, Guo SY (2011). Meta-analysis of clearing heat and removing toxicity therapy on ischemic stroke. *Pharmacology and Clinics of Chinese Materia Medica*.

[5] Chen X, Howard OM, Yang X, Wang L, Oppenheim JJ, Krakauer T (2002). Effects of Shuanghuanglian and Qingkailing, two multi-components of traditional Chinese medicinal preparations, on human leukocyte function. *Life Sciences*.

[6] Yue S, Li Q, Liu S (2006). Mechanism of neuroprotective effect induced by QingKaiLing as an adjuvant drug in rabbits with *E. coli* bacterial meningitis. *Acta Neurochirurgica*.

[7] Hua Q, Zhu XL, Li PT (2008). Refined Qing Kai Ling, traditional Chinese medicinal preparation, reduces ischemic stroke-induced infarct size and neurological deficits and increases expression of endothelial nitric oxide synthase. *Biological and Pharmaceutical Bulletin*.

[8] Lv L, Liu Y, Shi HF, Dong Q (2009). Qingkailing injection attenuates apoptosis and neurologic deficits in a rat model of intracerebral hemorrhage. *Journal of Ethnopharmacology*.

[9] Review Manager (RevMan) [Computer program] (2008). *Version 5 for Windows*.

[10] Tan F, Gu W, Huang T (2003). Effect of Qingkailing Injection on platelet CD62P and cytokines in patients with acute cerebral infarction. *Chinese Journal of Integrated Traditional and Western Medicine*.

[11] Zeng H, Feng P (2003). Clinical observation on 34 cases acute cerebral infarction with Qingkailing Zhusheye. *Journal of Emergency in Traditional Chinese Medicine*.

[12] Yang WX, Liu CQ, Kang JJ, Zhu JW, Xu SZ (2003). To study the therapeutic effectiveness of using high dose in the treatment of 30 patients with cerebral infarction. *Chinese Journal of the Practical Chinese with Modern Medicine*.

[13] Yan WP, Li YW (2010). Clinical observation on acute cerebral infarction with Qingkailing injectiion combined with low dose of urokinase. *Journal of Emergency in Traditional Chinese Medicine*.

[14] Wu XW, Chen B, Huang JB (2007). Effect of Qingkailing on the serum levels of TNF-*α* and IL-6 in patients with acute cerebral infarction. *Journal of Jingmen Technical College*.

[15] Liang SH (2000). Clinical observation on 40 cases acute cerebral infarction with Qingkailing Zhusheye. *Guangxi Medical Journal*.

[16] Yu BR, Liao YX (1999). Application of Qingkailing in treating cerebral infarction of large volume. *Chinese Journal of Rehabilitation Medicine*.

[17] Wu B, Liu M, Liu H (2007). Meta-analysis of traditional Chinese patent medicine for ischemic stroke. *Stroke*.

[18] Chen QT (1996). Diagnostic criteria and levels on and evaluation of neurological impairment for stroke patients. *Chinese Journal of Neurology*.

[19] Haldeman GA, Croft JB, Giles WH, Rashidee A (1999). Hospitalization of patients with heart failure: national hospital discharge survey, 1985 to 1995. *American Heart Journal*.

[20] Rosamond W, Flegal K, Furie K (2008). Heart disease and stroke statistics-2008 Update: a report from the American heart association statistics committee and stroke statistics subcommittee. *Circulation*.

[21] Hao Y, Kong X, Wu T (2010). Assessment of the safety of Qin Kai Ling injection: a systematic review. *Journal of Evidence-Based Medicine*.

